# Cellular rescue in a zebrafish model of congenital muscular dystrophy type 1A

**DOI:** 10.1038/s41536-019-0084-5

**Published:** 2019-11-15

**Authors:** T. E. Hall, A. J. Wood, O. Ehrlich, M. Li, C. S. Sonntag, N. J. Cole, I. G. Huttner, T. E. Sztal, P. D. Currie

**Affiliations:** 1 Australian Regenerative Medicine Institute, Monash University, Level 1, 15 Innovation Walk, Victoria, 3800 Australia; 2 Institute for Molecular Bioscience, University of Queensland, 306 Carmody Road, St Lucia, 4067 Australia; 3 Victor Chang Cardiac Research Institute, 405 Liverpool Street, Darlinghurst, Sydney, New South Wales 2010 Australia; 4 Anatomy & Histology, School of Medical Science, Anderson Stuart Building, Eastern Avenue, The University of Sydney, Sydney, New South Wales 2006 Australia; 5 Department of Biological Sciences, Monash University, Victoria, 3800 Australia; 6 EMBL Australia, Victorian Node, Monash University, Clayton, VIC 3800 Australia

**Keywords:** Diseases, Genetics

## Abstract

Laminins comprise structural components of basement membranes, critical in the regulation of differentiation, survival and migration of a diverse range of cell types, including skeletal muscle. Mutations in one muscle enriched Laminin isoform, *Laminin alpha2 (Lama2)*, results in the most common form of congenital muscular dystrophy, congenital muscular dystrophy type 1A (MDC1A). However, the exact cellular mechanism by which Laminin loss results in the pathological spectrum associated with MDC1A remains elusive. Here we show, via live tracking of individual muscle fibres, that dystrophic myofibres in the zebrafish model of MDC1A maintain sarcolemmal integrity and undergo dynamic remodelling behaviours post detachment, including focal sarcolemmal reattachment, cell extension and hyper-fusion with surrounding myoblasts. These observations imply the existence of a window of therapeutic opportunity, where detached cells may be “re-functionalised” prior to their delayed entry into the cell death program, a process we show can be achieved by muscle specific or systemic Laminin delivery. We further reveal that Laminin also acts as a pro-regenerative factor that stimulates muscle stem cell-mediated repair in *lama2*-deficient animals in vivo. The potential multi-mode of action of Laminin replacement therapy suggests it may provide a potent therapeutic axis for the treatment for MDC1A.

## Introduction

Laminins are integral structural components of the interstitial extracellular matrix and the basement membranes of animal tissues, providing mechanical stability and mediating interaction between cell surfaces. They are critically involved in such fundamental cellular processes as differentiation, survival and migration.^1,2^ Individual Laminin chains are assembled within the cell into cruciform,^3^ trimeric^4,5^ complexes, which are secreted through the plasma membrane onto its external surface.^6^ Each heterotrimer consists of one alpha, one beta and one gamma chain, such that each complex contains three separate gene products.^7^ Within the *laminin* gene family, heterogeneity within paralogous subunit genes allows the formation of a multitude of different combinations, each of which is associated with a particular expression domain or suite of cell types.^8,9^

Depending on the specific trimer involved, following secretion, the cruciform complexes then further self-assemble into a continuous, higher-order lattice, via interactions of the three short (N-terminal) arms.^10^ Such lattice building requires that the long arm, capped by the C-terminal globular domain of the alpha chain, is tethered to the cell surface.^11^ C-terminal globular domains bind the transmembrane dystroglycan complex (DGC),^12^ and, depending on the specific alpha chain involved, dimeric integrin receptors.^13^

Recessive mutations in individual laminin genes result in a considerable disease burden to society. Mutations in at least three different laminin chain genes result in junctional epidermolysis bullosa, *Laminin Gamma 3* (*LAMC2*),^14^ *Laminin Alpha 3* (*LAMA3*),^15^ *Laminin Beta 3* (*LAMB3*)^16,17^ and cutis laxa.^18^ Mutations in *Laminin Beta 2* (*LAMB2*) result in Pierson’s Syndrome.^19^ *Laminin alpha4* (*LAMA4*) mutations are associated with cardiomyopathy^20^ and mutations in *LAMA2* cause congenital muscular dystrophy type 1A (MDC1A).^21^

MDC1A is a severe, autosomal recessive disorder caused by genetic lesions in the human *Lama2* gene.^21–26^ The LAMA2 chain has been termed the “muscle specific chain”, and assembles with the beta1 and gamma1 chains to form the Laminin 211 (Lam211) complex, which is expressed in skeletal muscle cells, the placenta and Schwann cells surrounding peripheral nerve axons.^27^ Mice deficient in *Lama2* have a severe disease phenotype, characterised by progressive muscle atrophy, paresis of the limbs, cachexia and death at between 4 and 26 weeks.^28,29^ Histopathologically, the muscle is characterised by increased fibre size variation, pyknotic myofibre nuclei, mononuclear infiltration, and an increase in the area of interstitial tissue (both fibrotic and adipose). Although the muscle fibres are innervated appropriately, the proximal regions of some peripheral nerves show areas of dysmyelination,^30^ resulting in reduced conduction velocities.^31^ In addition, MDC1A patients often present with abnormal white matter morphologies in the central nervous system (CNS), although this is only infrequently associated with mental impairment.^23^ Symptoms primarily reflect the defects in skeletal muscle and peripheral nerves. When a human *lama2* encoding transgene under the regulation of a muscle-specific creatine kinase promoter was expressed within the Lama2-deficient *dy/dy* mouse,^28^ the phenotype was largely ameliorated, but the mice still retained a lameness attributed to dysmyelination.^32^ In addition, when *Laminin gamma1 (LAMC1)* was deleted specifically in myelinating Schwann cells, the mice also became lame^33^ reinforcing the notion that this aspect of the phenotype results specifically from loss of the Lam211 complex in these cells.

On a cellular level, the primary pathological consequence in both Schwann cells and muscle fibres appears to be loss of basement membrane surrounding individual cells, although the exact cellular basis of human pathology remains unclear. A dramatic reduction in BM staining has been documented in electron micrographs of either cell type from *dy/dy* mice.^34–36^ However, there are contradictory indications as to the effect of Laminin loss on the cells themselves. There is evidence that the primary muscle cell phenotype is caused by loss of adhesion. Counter-intuitively, mice and zebrafish deficient in Lama2 show less uptake of Evans blue dye, a marker of membrane permeability, into dystrophic muscle than *mdx* (Dystrophin-deficient) mice and zebrafish, despite a more severe tissue degeneration phenotype.^37–39^ Concomitantly, recent studies in the LARGE mouse^40^ have shown that when glycosylation of dystroglycan is disrupted, preventing Laminin binding, basement membrane forms appropriately but is not closely associated with the sarcolemma. As a result, in this model, contraction induces a "DMD-like" membrane fragility effect. Thus, exactly how loss of Laminin and its consequent binding to the dystrophin-associated glycoprotein and other membrane adhesion complexes results in congenital muscle muscular dystrophy remains far from clear.

Consequently, there are currently no directed treatment strategies that address the biochemical or genetic defects in MDC1A^41^ and there is an urgent need for more targeted treatment approaches. Recent studies in mice have indicated possible avenues for therapy including direct protein (as opposed to “gene”) replacement therapy of Laminin itself.^42–44^ This conceptually simple therapeutic approach has shown considerable promise in mouse models, but exactly how supplying exogenous Laminin to the dystrophic context of the MDC1A mouse model results in a suppression of dystrophic pathology remains unclear.

Gene therapy studies in the mouse, demonstrating restoration of the physical link between dystroglycan and the extracellular matrix (ECM) using an artificial mini-agrin linker^45–48^ have been partially successful, suggesting that restoring a structural linkage between the sarcolemma and the ECM in Lama2 deficiency is important. However, additional cell culture studies suggest a trophic requirement for laminin binding in maintaining muscle support,^49,50^ a property that has not been comprehensively examined.

In this study, we have used the optical clarity of the zebrafish larvae to examine the in vivo cell biological response to *lama2* deficiency and Laminin replacement therapy. We show that muscle fibre detachment occurs rapidly in Lama2-deficient muscle but detached fibres maintain an intact sarcolemma and are remarkably long lived as hyper-contracted fibres. Direct Laminin protein replacement prevented contraction-induced myofibre damage by supporting existing adhesions. However, in addition to this structural role, we also demonstrate that laminin replacement induces myofibre remodelling and survival in an adhesion-independent manner and promotes the reattachment of fibres to the ECM. Furthermore, Laminin replacement also induces a pro-regenerative response, stimulating muscle progenitor and stem cells both in dystrophic and muscle injury settings. These results suggest that Laminin replacement therapy has the potential ameliorate MDC1A pathology via number of possible non-overlapping mechanisms. Such insights into the effects of therapeutic strategies on the primary cellular pathology of *lama2* deficiency will be key to the development of targeted treatments for MDC1A treatment, which are currently lacking.

## Results

### Detached fibres are long lived and undergo extensive remodelling within *lama2*-deficient animals

**Fig. 1:**
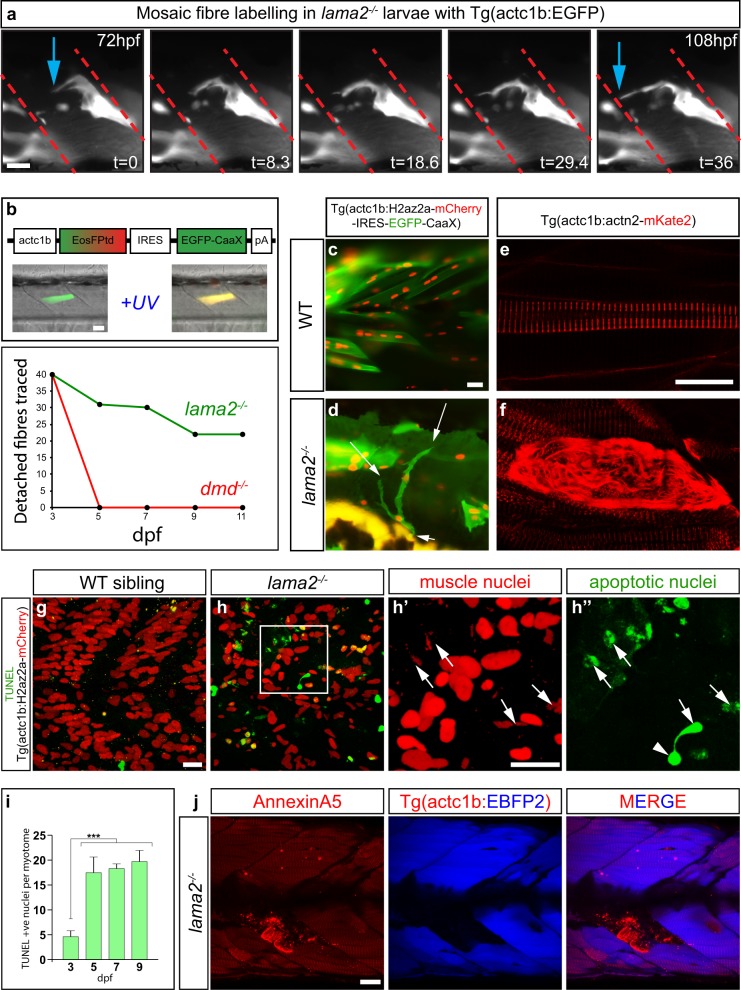
Dystrophic fibres in *lama2*^*−/−*^ larvae undergo extensive remodelling prior to undergoing cell death. **a** Still frames of continuous time-lapse analysis (Supplementary Movie 1) of mosaic labelled actc1b:GFP dystrophic fibres in lama2^−/−^ larvae reveals extensive remodelling and reattachment (blue arrows) to the matrix-rich regions of the vertical myosepta (red dashed lines). Time (*t*) is indicated in minutes. **b** Tracing of single dystrophic fibres in *lama2*^−*/−*^ and *dmd*^−*/−*^ larvae reveals *lama2*^−*/−*^ dystrophic fibres are long lived compared to those evident *dmd*^−*/−*^ animals. **c**, **d** Fibre remodelling is evident in dystrophic fibres in *lama2*^−*/−*^ larvae mosaically injected with an act1b:H2az2a-mCherry-IRES-EGFP-CaaX construct, which marks differentiated muscle nuclei in red and plasma membrane in green. This analysis identified extensive fibre branching (arrows, **d**) of an individual dystrophic fibre that was not evident in wildtype siblings similarly injected (**c**). **e**, **f** Fibres transgenically marked mosaically with the alpha-actinin-mKate2 fusion protein expressed shows the formation of new pre-myofibrils in the dystrophic context (**f**), compared to Z-line associated in the wildtype (**e**). **g**–**i** Dystrophic fibres in *lama2*^−*/−*^ animals undergo a delayed cell death. An increase in cell death is only seen at 5 dpf within the myotome of *lama2*^−*/−*^ larvae, several days after the onset of dystrophic pathology. **g**, **h″** TUNEL staining (Green) in wildtype sibling (**g**) and *lama2*^−*/−*^ larvae (**h**–**h″**) transgenic for Tg(actc1b:H2az2a-mCherry), which marks muscle nuclei in red, reveals the increase in cell death in muscle nuclei in *lama2*^−*/−*^. **h′** and **h″** are high magnification views of the region marked in **h**. **i** Quantitation of TUNEL-positive muscle nuclei in the myotomes of *lama2*^−*/−*^ animals. Significance is tested in a one-way ANOVA, Tukey’s test; ***p < 0.001. Results are expressed as a mean ± SD. **j** Secreted annexin5-mKate2 (red, arrows), which marks cells undergoing cell death, is specific for regions of fibre loss in 5 dpf larvae. Blue marks intact muscle fibres in Tg(actc1b:EBFP2) larvae. Scale bars, 20 μm

To examine the response of individual dystrophic fibres in the larval MDC1A zebrafish model, we used transgenic mosaic labelling of individual differentiated muscle fibres and the optical accessibility of living muscle tissue in the zebrafish system. This allowed us to trace the behaviour of dystrophic fibres in real time and to catalogue the length of time of fibre survival post basement membrane detachment. To indelibly identify individual fibres within each larva, we injected a construct that expressed GFP from the muscle-specific alpha actin promoter (actc1b) and under took continuous time-lapse photomicroscopy of individual dystrophic muscle fibres over a 36-h period from 3 days post-fertilisation (dpf) (Fig. 1a). This analysis revealed that these post-mitotic cells undergo extensive remodelling, extending protrusive processes that are able to re-establish contact with the basement membrane connective tissue (Fig. 1a and Supplementary Movie 1). Furthermore, all dystrophic fibres that were traced in this manner survived for the full period of the time-lapse analyse.

These observations suggested that dystrophic fibres within the *lama2*-deficient model can survive for a surprisingly lengthy period post-detachment from the basement membrane.

Since our time-lapse photomicroscopy was technically limited to periods of <2 days and all the fibres we examined survived for this period, we developed an individual fibre tracing technique the photoconvertable protein EosFPtd expressed again from the muscle specific alpha actin promoter. Photoconversion from green to red indelibly marks individual dystrophic fibres, allowing larvae to be maintained under free swimming conditions in between periods of observation and tracing of specific fibres for several days (Fig. 1b). By fusing the photoconvertible protein to the highly stable histone H2az2a, which binds directly into the chromatin, we were able to extend the timeframe of these experiments to trace the behaviour of the same individual dystrophic fibre up to 8 days post-photoconversion (11 dpf). We used this approach to label individual fibres both in the dystrophin-deficient line (*dmd*^*ta222a*^) and in the *lama2*-deficient MDC1A model (*lama2*^*teg15a*^) to compare the mode of fibre loss between in the two models (Fig. 1b). Forty individual fibres were traced in 40 different mutant larvae, and this analysis surprisingly revealed that over half fully detached, hyper-contracted, muscle fibres survived the full 8-day imaging period (55%, *n* = 22) and that 77% (*n* = 17) of these surviving fibres managed to reattach to the basement membrane from which they originally detached. By contrast similar fate mapping analyses of detached fibres in the dystrophin-deficient *dmd*^*ta222*^ line revealed that none of the 40 detached fibres examined were present at the next imaging time point 1 day post-conversion. This analysis revealed that in contrast to dystrophic fibres in dystrophin-deficient zebrafish, dystrophic fibres in the MDC1A model are long lived.

We next examined in more detail the nature of the remodelling that dystrophic fibres undergo by using more specific cellular markers. We labelled dystrophic fibres in *lama2*^−*/−*^ larvae mosaically injected with an actc1b:H2az2a-mCherry-IRES-EGFP-CaaX construct, which marks differentiated muscle nuclei in red and plasma membrane in green (Fig. 1c, d). So extensive can the remodelling of dystrophic fibres in *lama2*^*−/−*^ mutants become, mosaic transgenic fibres can be seen to split almost in two with separate “branches” of individual fibres attempting to attach to very separate regions of the myotomal ECM, often extending against the normal fibre orientation of surrounding healthy fibres (Fig. 1d), a behaviour never seen in non-dystrophic controls (*n* = 25 mutant and *n* = 25 wildtype (WT) larvae observed). Next we mosaically expressed mKate2-tagged alpha-actinin2 (actn2) which, in developing muscle fibres, first assembles into non-striated premyofibrils, before being stably incorporated into mature Z-lines in a characteristic banding pattern. This analysis showed that the remodelling of dystrophic fibres includes the formation of new, non-striated alpha-actinin-positive pre-myofibrils, suggesting the re-initiation of sarcomere assembly in dystrophic fibres (Fig. 1e, f) (*n* = 25 mutant and *n* = 25 WT larvae observed).

We then examined if there were any detectable levels of the activation of the cell death pathway within detached fibres to determine the nature of fibre loss in the zebrafish MDC1A model. To do this analysis, we crossed *lama2*^−/−^ animals to a transgenic line that expressed a nuclear localised version of mCherry from a muscle specific promoter. We then compared the levels of terminal deoxynucleotidyl transferase dUTP nick end labelling (TUNEL) staining within muscle nuclei of *lama2*^−/−^ mutant and sibling larvae (Fig. 1g–i). This analysis revealed that although the cell death pathway can be initiated in detached fibres in *lama2*^*−/−*^ muscle cells its incidence increases dramatically and plateaus several days after the initiation of pathology within the myotome of *lama2*^*−/−*^, at 3 dpf. This result was confirmed by an analysis of a newly generated transgenic line (Tg(actb2:secAnnexin5-mKate2) where the expression of AnnexinA5, which is specifically localised to apoptotic cells, is fused to the red fluorescent protein mKate2. Crossing this line into the *lama2*^*−*^^*/−*^ background revealed relatively few labelled cells at 3 dpf with labelling increasing at 5 dpf (Fig. 1j) confirming the initial observations of delayed cell death revealed by TUNEL staining. Collectively, these results suggest that detached fibres in the *lama2*^*−/−*^ context are long lived, only initiating the cell death pathway after extensive attempts to remodel.

### Lama2-deficient dystrophic fibres maintain sarcolemmal integrity and undergo significant remodelling post-detachment

**Fig. 2:**
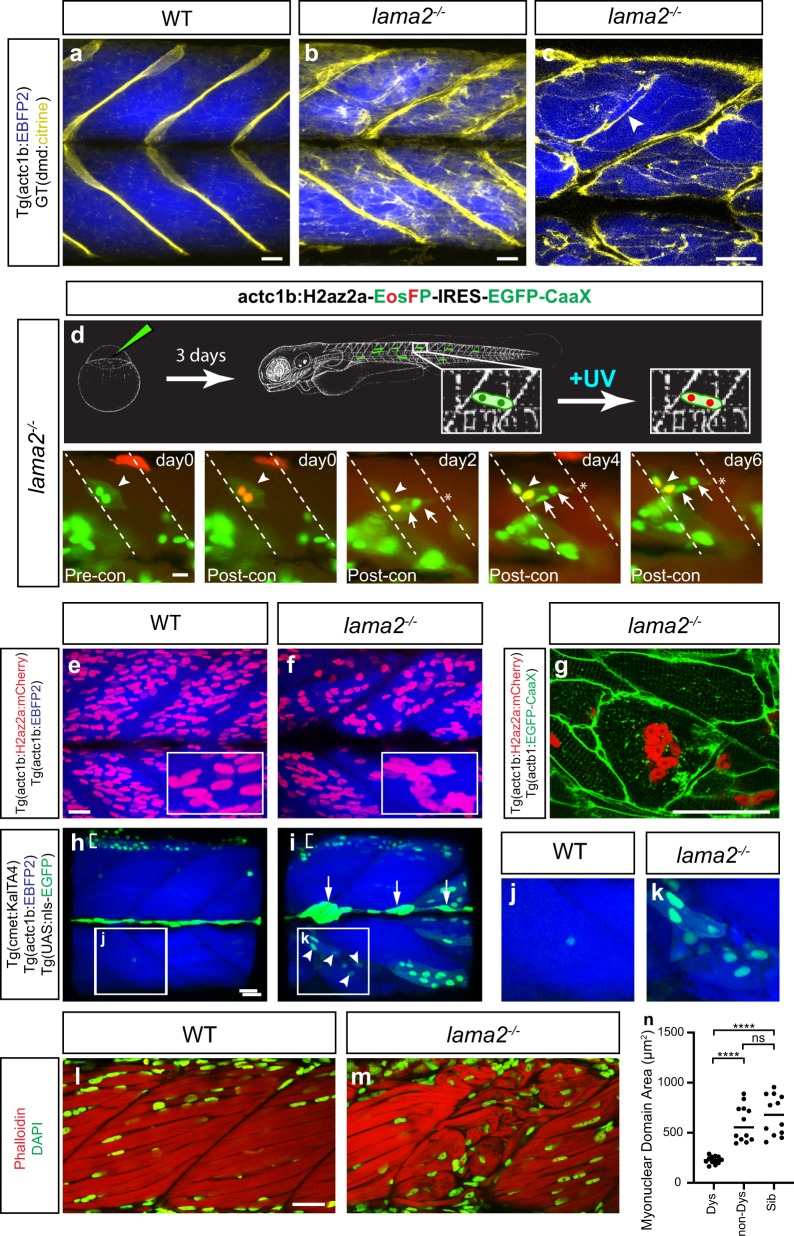
Long-lived dystrophic *lama2*^*−/−*^ fibres maintain fibre polarity, upregulate Dystrophin and undergo hyper-fusion. **a**–**c** Dystrophin expression (yellow) in the line GT(dmd-citrine), is mislocalised in *lama2*^*−/−*^ mutants (**b**) compared to WT (**a**) in confocal projections. Single confocal section (**c**) reveals junctional polarity of Dystrophin is maintained in *lama2*^*−/−*^ fibres (indicated by arrowhead). Muscle is marked in blue by BFP from Tg(actc1b:EBFP2). **a**, **b** Maximum projections from Supplementary Movies 2 and 3. **d** *lama2*^*−/−*^ dystrophic fibres undergo fusion during remodelling. Injection of actc1b:H2az2a-EosFPtd-IRES-EGFP-CaaX into *lama2*^−*/−*^ embryos allows photoconversion of muscle nuclei expressing H2az2a-EosFPtd from green to red at 3 dpf (arrrowheads). Fibres are tracked and addition of new nuclei (green, arrows) determined. This reveals fusion which precedes reattachment of the fibre to the opposite vertical myoseptum. Time is expressed as days post-photocoversion. **e**–**k** Hyper-fusion in *lama2*^−/−^ dystrophic fibres. **e**, **f** Maximum projections of WT sibling (**e**) and *lama2*^−/−^ (**f**) larvae transgenic for Tg(actc1b:H2az2a-mCherry) and Tg(actc1b:EBFP2), which mark muscle nuclei (red) and muscle (blue), respectively. Insets reveal the regular arrays of nuclei in WT are perturbed and clumped in *lama2*^*−/−*^ fibres. **g** Confocal section of dystrophic fibres within a *lama2*^*−/−*^ mutant transgenic for Tg(actc1b:H2z2a-mCherry) and Tg(actc1b:EGFP-CaaX). Hyper-fused fibres are evident, with the centrally located fibre exhibiting clumped nuclei, a situation never seen in wildtypes. **h**–**k** Activation of the stem cell compartment as a source of muscle nuclei. WT (**h**, **j**) or *lama2*^*−/−*^ (**i**, **k**) larvae transgenic for Tg(cmet:KalTA4; UAS-nlsGFP) and Tg(actc1b:EBFP2). Green marks the cmet-positive satellite cell compartment (54) and blue differentiated fibres, revealing the GFP-positive satellite cell-derived nuclei, specifically in *lama2*^*−/−*^ dystrophic fibres (arrowheads **i**, **k** inset). Arrows mark the lateral line and brackets indicate the cell body nuclei of the dorsal motor neurons, which also express cmet at high level (**h**, **i**). All panels of 3 dpf larvae unless otherwise denoted. **l**–**n** Quantitation of the hyper-fusion phenotype in *lama2*^*−/−*^ mutant larvae. Phalloidin (red) marks muscle fibres and DAPI (green) marks nuclei in wildtype sibling (**l**) and mutant larvae (**m**). Quantitation of the myonuclear domain size, which measures that relative amount muscle cell cytoplasm to muscle nuclei within individual fibres (**n**). More nuclei per unit area is represented by a smaller myonuclear domain size. Significance is tested in a one-way ANOVA, Tukey’s test; ****p < 0.0001. Scale bars, 20 μm

We examined the integrity of the sarcolemma using a number of distinct transgenic lines. To visualise the myofibre cytoplasm, the transgenic line Tg(actc1b:EBFP2)^pc5^ (ref. ^51^) was crossed to the fliptrap line GT(dmd-citrine)^ct90a^ which results from the fluorescent protein, citrine, being incorporated into the endogenous dystrophin gene, therefore generating full-length endogenous Dystrophin-Citrine fusion protein.^52^ As a component of the DGC, dystrophin associates with the cytoplasmic face of the muscle sarcolemma and is strongly expressed at the ends of muscle fibres, adjacent to the myotendinous junction (MTJ). The zebrafish lines Tg(actc1b:EBFP2)^pc5^ and GT(dmd-citrine)^ct90a^ were crossed onto the lama2^*−/−*^ mutants and their progeny imaged at 5 dpf (Fig. 2a–c, Supplementary Movies 2, 3). Dystrophin expression is mis-localised in *lama2*^−*/−*^ mutants, with detached muscle fibres (arrowhead) expressing high levels of Dystrophin protein at the junctional ends of detached muscle cells (Fig. 2c). This redistribution occurs in the absence of an upregulation of *dystrophin* transcription, as determined by qPCR analysis *lama2*^*−/−*^ mutant and WT siblings (Supplementary Data Fig. 2). This indicates that although a fibre has lost contact with the ECM to which it attaches it still maintains fibre polarity and likely an intact junctional DGC complex, since Dystrophin is nearly always lost from the sarcolemma in contexts where when the DGC is disrupted. In line with the results above, sarcolemmal integrity is also indicated by retention of BFP within the cytoplasm of detached fibres.

### Hyper-nuclear fusion contributes to remodelling in *lama2*-deficient animals

Our nuclear-localised fibre-tracing construct was conceived so as to increase the specificity of conversion to a single point using a pinhole on a standard compound fluorescence microscope, and allow better resolution to our photoconversion. However, the nuclear localisation of the fluorescent signal produced the unexpected result that fibres undergoing remodelling frequently gained non-photoconverted (green not red) nuclei (45% of detached fibres traced *n* = 40, Fig. 2d). Since differentiated muscle fibres are post-mitotic syncytia, our interpretation of these data was that the new nuclei observed alongside existing photoconverted nuclei arose from fusion events with muscle progenitor cells, the existence of which we have recently documented in retrospective clonal analyses.^53^ Dystrophic fibres can gain multiple nuclei over the course of the timeframe of analysis and exhibit a dramatic “hyper-fusion” phenotype highly similar to that displayed when components of the muscle fusion pathway are ectopically activated in muscle cells^54^ (Fig. 2d–g, Supplementary Movie 4). To determine the source of cells that provided nuclear fusion to the dystrophic fibres, we examined the expression of the double transgene Tg(cmet:KalTA4-T2A-mCherry; UAS-nlsGFP) in WT and *lama2*^*−/−*^ larvae. In this line, KalTA4-T2A-mCherry marks the cmet-positive muscle stem cell compartment or satellite cells,^53^ and nuclear GFP is driven in the same cells by the KalTA4 transgene. This analysis revealed the fusion of GFP-positive, satellite cell-derived, nuclei occurs specifically in *lama2*^*−/−*^ dystrophic fibres (Fig. 2h–k, Supplementary Movies 5, 6) indicating that satellite cells are a source of hyper-fusion in dystrophic fibres. In order to quantify hyper-fusion in a meaningful and systematic manner, we calculated the myonuclear domain size for intra-myofibrillar nuclei within dystrophic areas of *lama2*^−*/−*^ myotomes, non-dystrophic areas of *lama2*^*−/−*^ myotomes and within sibs (Fig. 2l–n). These data clearly show that dystrophic fibres have a significantly greater number of nuclei per unit area.

Collectively, our studies reveal that detached fibres within our congenital muscular dystrophy model undergo fusion events, remodel their sarcolemma and reattach to existing areas of matrix; processes and pathways that are not normally activated in non-dystrophic fibres. These observations suggest that dystrophic fibres in the zebrafish MDC1A model activate pro-survival pathways in response to detachment from the muscle basement membrane, a process that if augmented could lead to the prevention of the activation of cell death pathways in individual dystrophic fibres.

### Muscle specific or systemic laminin protein replacement ameliorates the *lama2*^*−/−*^ phenotype

The long-lived nature of the detached fibres and the extensive remodelling that occurs after fibre detachment suggested that if attachment and survival of fibres could be influenced in a pro-survival manner there was a possibility that detached fibres could be “refunctionalised”. One possible route to achieving this goal is by Laminin replacement therapy, with our results suggesting that re-functionalisation of dystrophic fibres could be one mechanism by which this therapy may occur. Previous studies have suggested that intramuscular injection can protect muscles of both lama2-deficient and dystrophin-deficient mice from physical damage induced by eccentric contraction^42,55^ as can transgenic expression in both these models, but the re-functionalisation of detached fibres has not been demonstrated in these contexts.

**Fig. 3:**
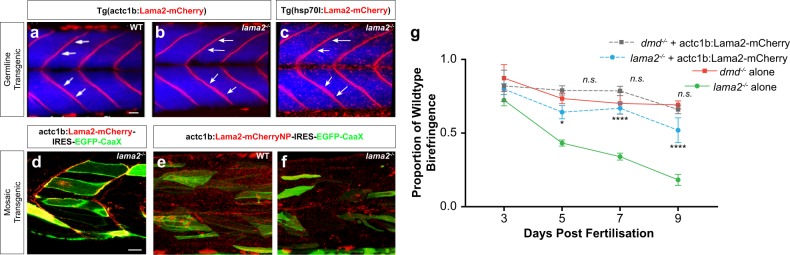
Transgenic delivery of laminin alleviates dystrophic pathology in *lama2*^*−/−*^ animals. **a**, **b** Germline transgenic larvae expressing Tg(actc1b:Lama2-mCherry) results in correct Lama2-mCherry (red) deposition at the myosepta in WT (**a**) and *lama2*^*−/−*^ deficient (**b**) larvae and rescues the dystrophic pathology in *lama2*^*−/−*^ animals. **c** Germline transgenic *lama2*^−*/−*^ larvae carrying the Tg(hsp70l:Lama2-mCherry) cassette are rescued upon heat-shock-induced expression of the Lama2-mCherry fusion protein (red). Blue marks differentiated muscle fibres in the Tg(actc1b:EBFP2). The focal “dot-like” Lama2-mCherry localisation results from the non-tissue-specific heat shock induction strategy, which also induces expression in non-myotomal cells. In this image, non-target expression is most prominent in cells of the epidermis. **d**–**f** Mosaic overexpression of Lama2 can rescue pathology in *lama2*^−/−^ larvae. Injection of actc1b:Lama2-mCherry-IRES-GFP-CaaX into *lama2*^*−/−*^ (**d**) reveals that even a low level of mosaic transgenesis (secreting transgenic cells labelled by localisation of GFP to the membranes) results in correct localisation to the myosepta, and a rescue of pathology (**g**). By contrast a mutant, non-polymerising (NP) form of Lama2, when expressed in the same manner (actc1b:Lama2-NPmCherry-IRES-GFP-CaaX), fails to accumulate at the myosepta in WT larvae (**e**) and does not rescue *lama2*^−*/−*^ mutants (**f**). **g** Injection of actc1b:Lama2-mCherry-IRES-GFP-CaaX into *dmd*^−/−^ larvae does not rescue muscle integrity. By contrast similar experiments using *lama2*^−/−^ larvae results in an amelioration of muscle pathology. Results are expressed as a mean ± SD, each group *n* = 12 per data point. Significance is tested in a two-way ANOVA between groups at each time point; *p < 0.05, ****p < 0.0001. Scale bars, 20 μm

As an initial proof of principle, we overexpressed lama2 within the *lama2*^*−/−*^ mutants in germline transgenic animal from the muscle specific alpha actin promoter (Fig. 3a, b). As lama2 is also expressed in peripheral nerves, it has been postulated that some aspects of the human muscle pathology may result from a neuropathy. Our analysis confirmed the ability of Lama2 driven from the muscle specific promoter to completely rescue the pathology of *lama2* mutants that were viable and fertile as adults (Fig. 3a, b, *n* = 10) confirming that, at least in the zebrafish MDC1A model, that the primary pathology results from a disruption of muscle integrity.

We also wished to extend this result using a universal overexpression strategy as it remained possible that overexpression of laminin more broadly within an embryo could have a detrimental effect on tissue development. Furthermore, we wished to be able to add back, in a temporally controlled fashion, the expression of Laminin to diseased muscle at different time points during the progression of dystrophic pathology in our mutants. In order to undertake such an approach, we cloned the lama2–mCherry fusion open reading frame downstream of the HSP70l heat shock promoter and generated germline transgenic animals. We then crossed this transgene into the background of the *lama2*^*−/−*^ animals. Heat shock during embryonic development generated a similar level of Lama2-mCherry expression within the myosepta of mutant animals to that generated with muscle specific over-expression (compare Fig. 3c to a and b). In the heat shock promoter driven line, additional labelled Lama2-mCherry is present within non-muscle cells, in line with the global nature of the heat shock. Dermal cells, most apparent ventrally and on the surface of the myotome, express high levels of Lama2-mCherry (Supplementary Movies 7–9). Intriguingly, despite the global nature of the heat shock, which would activate *lama2* expression in every cell, Lama2-mCherry was correctly located to the MTJ, the sight of embryonic and larval muscle attachment (Fig. 3c). This finding is in line with our previous observation that Lama2, secreted from individual muscle fibre clones, or from surrounding tissues, can sustain a partial, non-cell autonomous rescue, of muscle integrity in *lama2*-deficient myotomes.^56^ Furthermore, global heat shock in this line at 24 hours post-fertilisation (hpf), 3 dpf, 7 dpf, and then every week until adulthood was able to fully rescue the phenotype of *lama2* mutant fish, which could now survive to adulthood, were fertile and possessed no overt pathological signs of dystrophy (*n* = 17).

Next, we sought to determine how Laminin overexpression results in a rescue of dystrophic pathology at a cellular level. Using mosaic analysis, we expressed an mCherry-tagged lama2 chain from secreting muscle cells, which were labelled with a membrane-localised GFP. This simultaneously allowed us to visualise the secretion of laminin directly and also identify the cells from which it was secreted. Consistent with our previous studies^56^ and the observations made above, we observed that laminin was secreted appropriately from transgenic cell clones and accumulated at the junctional sarcolemma of the muscle basement membrane (Fig. 3d). Furthermore, despite the sporadic nature of the cell clones within the muscle, labelled laminin was distributed in a continuous seam at the muscle basement membrane, often many cell diameters from the nearest secreting cell. When we quantified the level of birefringence (which quantitates polarised light refraction generated as an intrinsic property of muscle and hence can provide a quantitative measure of muscle integrity^56^) in mosaically injected fish, we identified a significant rescue of pathology that could not be generated by mutant non-polymerisable forms of laminin, which did not localise to the basement membrane in WT or *lama2*^−*/*−^ larvae (Fig. 3e, f). This result suggests that it is likely to be the matrix interaction and stability that is important to laminin function in this context rather than specific signalling properties activated by secreted laminin itself.

To test whether laminin replacement protects muscle fibres from adhesion failure, we compared the loss of birefringence over time between animals mosaically expressing lama2-mCherry and non-expressing controls. The loss of birefringence in the lama2-mCherry-expressing *lama2*^*−/−*^ mutants was lower than the loss in the control animals, indicating that replacement of lama2 protects muscle from contraction-induced damage (Fig. 3g). By contrast, we could see no rescue of the birefringence deficits of similarly injected *dmd*^−*/−*^ larvae, suggesting that mosaic transgenic delivery of Laminin was not sufficient for pathological rescue in animals lacking Dystrophin.

### Laminin expression post detachment can lead to refunctionalisation of detached muscle fibres in *lama2*^*−/−*^ mutants

We next wished to examine if providing Laminin back to the *lama2*^−*/−*^ environment could stimulate fibre survival and reattachment of already detached fibres. This would directly test if Laminin therapy could stimulate refunctionalisation of dystrophic fibres in a model of congenital muscular dystrophy. To undertake this analysis, we used the heat shock line we had generated to induce timed expression of lama2 back to deficient animals and assayed the survival of detached dystrophic fibres and their behaviour.

**Fig. 4:**
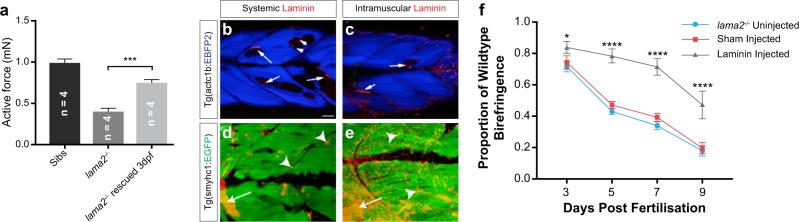
Laminin can rescue pathology in *lama2*^−/−^ larvae. **a** Heat shocked transgenic *lama2*^−*/−*^ larvae carrying the Tg(hsp70l:Lama2-mCherry) cassette are were measured for active force at 6 dpf using a force transducer. The optimal length for active contraction was first determined, then muscles were stimulated at this length at supra-maximal voltage for maximal force production. Providing heatshock at 3 but not at 5 dpf resulted in an increased active force within mutants. **b**, **c** Two distinct methods of exogenous delivery of fluorescently labelled Lam111, systemic (**b**) and direct intramuscular injection (**c**) lead to the accumulation of laminin protein preferentially at areas of fibre loss (arrows), as detected in Tg(actc1b:EBFP2) as reduced blue fluorescence. **d**, **e** Injection into the transgenic line Tg(smyhc1:GFP) which provides single-fibre resolution of the superficial slow muscle palisade, reveals both focal (arrowheads) and large fibre-associated regions (arrows). **b**, **c** Single confocal plane images; **d**, **e** maximum projection images of myotome. Larvae at 5 dpf. **f** Injection of Lam111 at 3 dpf ameliorates the rate of muscle loss in *lama2*^−/−^ larvae. Results are expressed as a mean ± SD, each group *n* = 12 per data point. Significance is tested in a two-way ANOVA between groups at each time point; *p < 0.05, ****p < 0.0001. Scale bars, 20 μm

We traced fibre reattachment events in heat shock versus non-heat shock *lama2*^−*/−*^ using the same procedure we describe above. In this approach, we heat shocked at 3 dpf, a period when the dystrophic pathology was fully evident and examined fibre survival, reattachment, fusion and death every 2 days until 11 dpf. This analysis revealed that there was a significant increase in fibre survival and attachment of dystrophic fibres (untreated 55% survival *n* = 40, 3 dpf HS 85% survival *n* = 40, Tukey's test, *p* < 0.0036). We also assayed the physiological rescue that heat shock at different stages (3 dpf and 5 dpf) could provide to *lama2*^−/−^ muscle. Heat shocked and control animals were measured for active force at 6 dpf using a specialised force transducer (Fig. 4a). The optimal length for active contraction was first determined, then muscles were stimulated at this length at supra-maximal voltage for maximal force production. For analysis, the active force measured at each pulse duration was normalised to the active force at 5 ms, which was the maximal. This analysis revealed that providing heat shock at 3 dpf resulted in an increase active force within mutants indicating that a period of recovery post heat shock is necessary to influence the re-functionalisation of individual muscle fibres.

Next, we examined if injection of exogenously supplied laminin could rescue the pathology of *lama2*^*−/−*^ larvae when administered after the onset of the dystrophic pathology. In order to visualise the localisation of the laminin protein treatment within the skeletal muscle, the previously described transgenic lines, Tg(actc1b:EBFP2)^pc5^ and Tg(smyhc1:EGFP), were crossed onto the *lama2*^*−/−*^ mutant and the progeny were observed. Laminin treatment was administrated 3 dpf, a point at which, as described above, the *lama2*^*−/−*^ mutant phenotype exhibits fully penetrant myotomal lesions.^38^ Two different methods were tested. Initially fluorescently tagged laminin 111 (lam111) was injected systemically into the circulation through injection in the pericardial sinus. Structurally, lam111 is most similar to the lam211 complex absent in MDC1A patients and is able to functionally substitute for lam211 in a number of assays.^42,43^ Consequently, both the exogenous application and the upregulation of the endogenous expression of lam111 are being targeted therapeutically in MDC1A, a process that would also likely side step the immunological barrier of supplying lam211 to lama2-deficient patients.^42,43,57^ Furthermore, unlike lam211, purified and functionally validated sources of lam111 are readily available. This injection resulted in the localisation of laminin protein into the muscle and was often associated with detached fibres (Fig. 4b, d). A second approach of direct intramuscular injection was also trialed. In this method, lam111 was injected within skeletal muscle above the anal pore as this could be used as an internal marker to ensure that was consistently delivered within the larval zebrafish. Using this procedure, 1 nl of the laminin protein intramuscularly in 3 dpf zebrafish, at the concentration of 250 μg/ml, and also localised to the regions of fibre loss (Fig. 4c, e). Furthermore, injection in this manner resulted in a significant amelioration of the skeletal muscle integrity defect evident in these mutants (Fig. 4f). Collectively, these results indicate that application of Laminin to dystrophic muscle fibres can be used to promote fibre survival.

### Laminin also acts as a pro-regenerative factor that stimulates stem cell-mediated repair in *lama2*-deficient animals

**Fig. 5:**
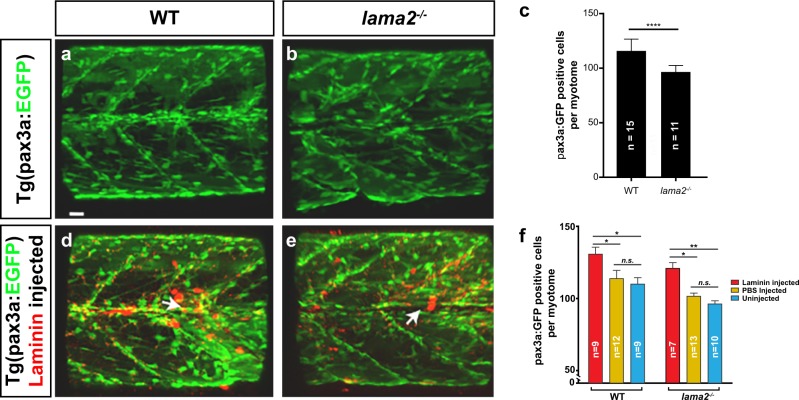
*lama2*^*−/−*^ mutant larvae possess defects in muscle stem and progenitor cell formation that are rescued by Laminin protein injection. **a**–**c** The transgenic line TgBAC(*pax3a*:GFP) marks muscle stem and progenitor cells in zebrafish larvae. *lama2*^*−/−*^ mutants (**b**) possess reduced numbers of pax3a-GFP-positive cells compared to WT (**a**), quantitated in **c** (Results expressed as mean ± SD. For each group, *n* ≥ 11. Statistics: *t*-test, two-tailed; ****p < 0.0001). **d**, **e** Intramuscular injection of fluorescently labelled Laminin protein (red) into *lama2*^*−/−*^ mutants (**e**) rescues pax3a-GFP-positive cell numbers, and increases them significantly over WT levels (**d**), quantitated in **f** (Results expressed as mean ± SD. For each group, *n* ≥ 7. Statistics: one-way ANOVA, Tukey’s test; *p < 0.05, **p < 0.01). Scale bars, 20 μm

Another mechanism by which laminin treatment may influence *lama2*^*−/−*^ CMD pathology is through its role as a component of the muscle stem cell niche. In order to examine the zebrafish muscle stem cell compartment, the transgenic line TgBAC(pax3a: GFP)^58^ was used. This line marks both the stem and progenitor cell compartment during muscle regeneration and provides a broad cellular assay of activation of the regenerative response in vivo.^53,58^ The muscle stem and progenitor cell compartments of *lama2*^*−/−*^ and sibling myotomes were examined at 6 dpf and revealed a significantly reduced number of pax3a GFP-positive cells in the *lama2*^*−/−*^ model (Fig. 5a–c) indicating a direct defect in the muscle stem cell niche in the lama2-deficient context.

We next examined if restoring laminin to the muscle of *lama2*-deficient animals, using the methods we describe above, would restore muscle stem and progenitor cells levels. In order to examine the lam111 proteins effect on the muscle stem cell compartment, TgBAC(pax3a:GFP) *lama2*^−*/−*^ zebrafish were treated with lam111 protein at 3 dpf and examined 3 days post-injection (Fig. 5d, e, *n* ≥ 7 per treatment). Treatment increased the number of muscle stem cells surrounding the site of delivery (white arrow Fig. 5e) and significantly increased the muscle stem cell population (Fig. 5f) rescuing the stem cell deficits evident in *lama2*^*−/−*^ larvae. Surprisingly, we also observed that laminin treatment produced an effect on the pax3-GFP cells of sibling larvae, significantly increasing the number of muscle progenitor cells per myotome (Fig. 5d, f). These results suggest that lam111 treatment is pro-regenerative, even in the absence of pathology.

### The addition of exogenous laminin within skeletal muscle alters the muscle stem cell niche and stimulates integrin signalling

In order to further examine the effect of laminin treatments within the skeletal muscle, we attempted to dissect the nature of laminin-mediated cell signalling. Focal adhesion kinase (FAK) localisation, a downstream effector of integrin signalling, can be used to identify when and where laminin-integrin-FAK interactions are occurring. FAK becomes phosphorylated when it interacts with integrin and phosphorylated-FAK (p-FAK) can only interact with activated integrin.^59,60^ Integrin becomes activated through conformational changes that occur when it interacts with its ligands, including laminin. By observing the expression levels of p-FAK at the myoseptum, we are therefore able to infer whether the introduction of exogenous laminin protein has increased integrin-mediated cell signalling within skeletal muscle.

**Fig. 6:**
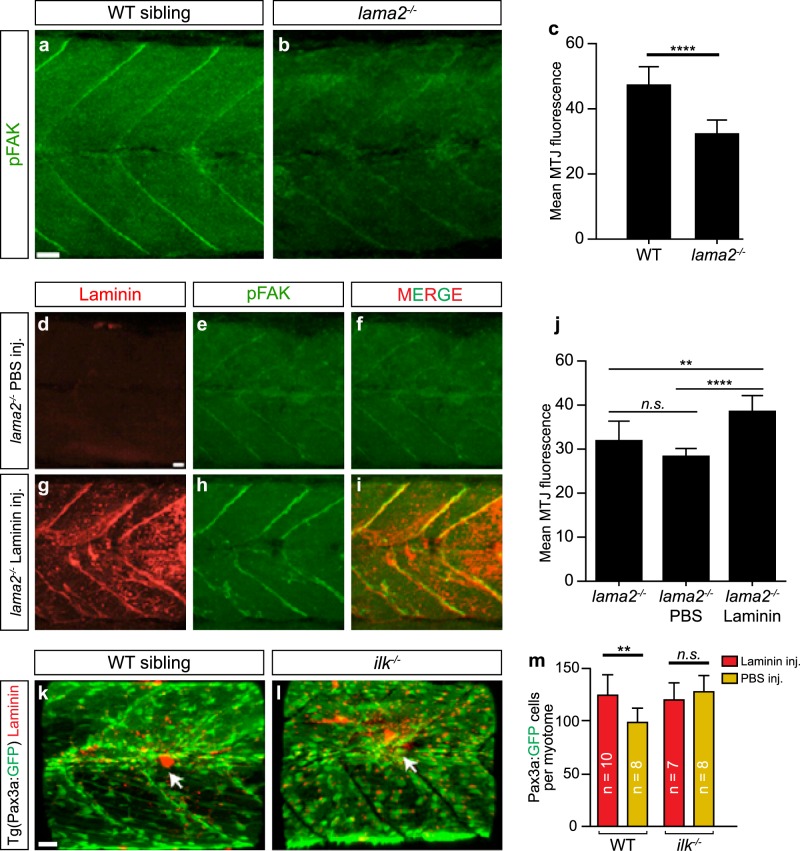
Laminin stimulation of stem cell proliferation requires downstream integrin signalling. **a**, **b** Representative images of phosphorylated-FAK expression (green) within skeletal muscle of 6 dpf lama2^−/−^ or sibling zebrafish. Scale bar, 20 μm. **c** Quantification of expression levels of p-FAK at the myotendious junctions of 6 dpf *lama2*^*−/−*^ or sibling zebrafish. Results are expressed as mean ± SD. Statistics: *t*‐test, two tailed; *p < 0.05, **p < 0.01, ***p < 0.001, ****p < 0.0001. **d**–**i** Representative images of p-FAK expression (green) in 6 dpf *lama2*^*−/−*^ zebrafish treated with laminin protein (red) or PBS. Note the colocalization of both these proteins at the myotendious junctions. Scale bar, 20 μm. **j** Quantification of expression levels of p-FAK at the myotendious junctions of 6 dpf *lama2*^*−/−*^ zebrafish that were either treated with exogenous laminin, PBS (orange) or untreated. Results are expressed as a mean ± SD. Statistics: one-way ANOVA, Tukey’s test; *p < 0.05, **p < 0.01, ***p < 0.001, ****p < 0.0001. **k**, **l** Representative images of Laminin treatment in the *ilk*^−*/−*^ mutant. White arrow indicates where intramuscular injection took place. Muscle stem cells marked by TgBAC(pax3a:GFP). Scale bar, 20 μm. **m** Quantitative analysis shows there is no significant effect on the *ilk*^−*/−*^ mutant when laminin treated; *ilk*^*−/−*^ mutant zebrafish were either treated with exogenous laminin or PBS. Results are expressed as a mean ± SD. For each group, *n* ≥ 6. Statistics: one-way ANOVA, Tukey’s test; *p < 0.05, **p < 0.01. Scale bars, 20 μm

To undertake this analysis, p-FAK expression levels were assessed at the MTJs of non-treated *lama2*^−*/−*^ and sibling zebrafish, an analysis that revealed that p-FAK expression is significantly decreased in the *lama2*^−*/−*^ mutants (Fig. 6a–c) (*n* ≥ 4 per treatment). Lama2-deficient zebrafish at 3 dpf were injected with either laminin protein or PBS and then observed at 3 days post-injection, 6 dpf (Fig. 6d–j, *n* ≥ 4 per treatment). p-FAK quantification revealed a significant increase within the Laminin-treated mutant and WT sibling zebrafish compared with controls (Fig. 6d–j, *n* ≥ 4 per treatment).

Integrin linked kinase (ILK) is another downstream component of the integrin signalling pathway, required for the transduction of intracellular signals in which have already been generated.^20^ In order to determine if the laminin-integrin-ILK cell-signalling axis is required to stimulate the muscle stem cell population, laminin injections were performed into *ilk*^*−/−*^ mutants (Fig. 6k–m, *n* ≥ 7 per treatment). Despite the injection of Laminin eliciting the same pro-regenerative response described above in siblings, Laminin injection into *ilk*^*−/−*^ mutants failed to increase stem and progenitor cells indicating that the activation of stem cell proliferation occurs through Laminin stimulation of the integrin signalling pathway.

## Discussion

Our results show that dystrophic, hyper-contracted fibres in the zebrafish *lama2*^*−/−*^ congenital muscular dystrophy model are long lived, exhibit sarcolemmal integrity and undergo complex remodelling behaviours post detachment that include membrane protrusion and fibre branching. Perhaps the most surprising of our observations was that detached fibres underwent hyper-fusion, a unique property of the detached fibre. These observations indicate that dystrophic detached fibres activate specific molecular processes that are not evident within uninjured fibres. Taken together with our previously published observations on the upregulation of specific adhesion molecules in detached fibres,^56^ we suggest that specific survival pathways are activated within dystrophic fibres that stimulate fibre remodelling. These observations and the considerable longevity of fibres post-detachment, suggests that there may exist a window of time post-detachment of a dystrophic muscle fibre, in which “refunctionalisation” may be attempted, prior to its entry into the cell death program. We have sought to validate this approach using laminin replacement protocols and test, if “fibre refuctionalisation” presents as a realistically therapeutic option for MDC1A, at least in the context of the zebrafish model.

Using a variety of methods to overexpress Laminin in the dystrophic condition, we were able to show that Laminin supplied prophylactically could prevent fibre detachment, and this could be achieved at surprisingly low levels of mosaic transgenesis. Using a genetically tagged version of Laminin, we could monitor in real time how deposition of Laminin proceeded in this context and revealed that Laminin secreted from individual muscle cells could be deposited many cell diameters away from the expressing fibres, within the ECM-rich region of the myosepta. These results provide some support for the idea that gene therapy approaches for Laminin may have a relative low hurdle to overcome in terms cellular infection rates in order to generate efficacy. We could further show that Laminin supplied post-onset of dystrophic conditions could also ameliorate the pathological hallmarks of fibre loss. Importantly, we found that this effect could be achieved by the direct injection of Laminin protein 111 into the muscle mass itself. Thus, laminin replacement therapy can act to prevent dystrophic fibres forming in the first place and secondly to stimulate pro-survival pathways required for their refunctionalisation.

As Laminin forms part of the muscle stem cell niche of the satellite cell, we also explored if there was some influence of muscle stem and progenitor cells in *lama2-*deficient animals. Our recent work has suggested that the ECM-rich regions of the septa provide proliferative cues for activated stem cells and work on mammalian stem cells has suggested that regulation of the stem cell niche is a critical aspect of the progression of the dystrophic condition in the *mdx* mouse.^53,61^ These studies reveal that dystrophin is expressed and its localisation is polarised within the stem cell niche. Furthermore, mutations in *Dystrophin* lead to deficits in stem cell self-renewal indicating that Duchenne muscular dystrophy is also a stem cell disease. Laminin, as a central component of the muscle ECM and a ligand for the DGC expressed on satellite cells, is well placed to influence the muscle stem cell niche directly. That *lama2*-deficient zebrafish larvae show deficits in stem cell numbers that can be rescued by the injection of exogenous lam111 supports a role for Laminin in the regulation of stem cell dynamics. It also suggests that congenital muscular dystrophy may too result in a stem cell deficit that mirrors those evident in the *mdx* mouse model, a hypothesis supported by the stem cell activation we see in our zebrafish model.

Surprisingly, Laminin injection was also pro-regenerative outside the context of *lama2* deficiency, increasing stem cell proliferation within injected siblings. These results suggest that the application of free laminin to muscle injury could be a viable approach to accelerate muscle repair and may be a useful avenue for inducing or bolstering regenerative capacities where they are supressed in contexts such as aging. This effect is modulated by activation of the integrin pathway, a specific set of receptors for Laminin binding on the muscle progenitor surface. Integrin signals have also recently been shown to be required for correct activation of the stem cell niche, with the core receptor component of b1-integrin being required to maintain muscle stem cell homoeostasis. Aged muscle shows a loss of b1-integrin sensitivity and its restoration increases the regenerative capacity of old muscle. Thus, it is possible that defects in Laminin polymerisation or integrity, and a loss of its ability to activate integrin signalling within muscle stem cells may underlie aspects of the aging stem cell niche.

Collectively, our studies suggest that Laminin replacement therapy could act at multiple points, utilising distinct pathways, to restore muscle function in congenital muscular dystrophy. The described stem cell activation axis has the potential to synergise with the “refunctionalisation” capacity that Laminin therapy may provide for dystrophic fibres, which in zebrafish *lama2* mutants display an extraordinary capacity to remodel. The long-lived nature of dystrophic fibres in the zebrafish model of MDC1A, and the differing cell biological outcomes for dystrophic fibres when compared to the dystrophin-deficient context, suggest that a highly distinct pathological process underlies fibre weakness in these systems. On a simpler level, we have shown that Laminin injection or restoration has the capacity to stabilise muscle attachments and prevent dystrophic fibre damage from progressing. Thus, Laminin has the capacity to act on three separate levels to restore muscle integrity in MDC1A; prevention of dystrophic fibre formation, refunctionalisation of dystrophic fibres and stimulation of muscle repair and regeneration in the context of disease.

## Methods

### Zebrafish lines

Previously described transgenic fish lines used were TgBAC(pax3a: GFP)^il50^ (ref. ^58^), Tg(actc1b:EBFP2)^pc5^ (ref. ^51^) and GT(dmd-citrine)^ct90a^ (ref. ^52^), Tg(actb2:EGFP-CaaX)^pc10^ (ref. ^62^), Tg(met:mCherry-2A-KalTA4)^pc24^ (ref. ^53^), Tg(actc1b:H2az2a-mCherry)^pc6^ (ref. ^56^). Mutant lines used were *lama2* (*caf*^*teg15a*^),^38^ *dmd* (*sap*^*ta222*^)^39^ and *ilk* (*loc*^*hu801*^).^20^ The transgenic lines Tg(actc1b:Lama2-mCherry), Tg(hsp70l:Lama2-mCherry), Tg(UAS-nlsEGFP) and Tg(actb2:secAnnexin5-mKate2) were made using the Tol2 transgenesis system via modular gateway cloning and generated plasmids injected into fertilsed embryos using previously published techniques.^53^

### Conditional rescue

The Tg(hsp70l:lama2-mCherry) transgene was first crossed into *lama2* mutant background. Heterozygous *lama2* zebrafish with the transgene were then genotyped and identified. Embryos from the heterozygotes incross were collected and heat shocked at 24 hpf, 3 dpf, 7 dpf and then every week until adulthood. Homozygous *lama2* mutant adults were identified by genotyping on fin-clips. For cell fate tracing experiments, the transgenic rescue was carried out at varied developmental stages (24 hpf, 3 dpf and 5 dpf) by incubating the larvae at 39 °C in E3 medium for 2 h.

### Birefringence

Birefringence was quantified as previously described.^56^

### Mosaic analyses

DNA constructs were injected at the 1-cell stage as previously described.^53^ Embryos were incubated until they had reached the appropriate stage of development, before being selected for an appropriate degree of mosaicism.

### Cell fate tracing and immunohistochemistry

The photoconvertable DNA constructs (actc1b-EoSFPtd-IRES-EGPFcaax and actc1b- H2az2a-EoSFPtd) were injected into embryos at the 1-cell stage. To initiate cell fate tracing at 3 dpf, the larvae were anaesthetised and mounted in silicone cast mould with E3 medium, and single cells/nuclei were then converted using blue channel fluorescence through a pinhole aperture and ×60 immersion objective for 3 min. The photoconversion result of individual fibres was monitored under immediately after the UV exposure and the fate of individual fibres determined at 5, 7, 9 and 11 dpf. For observation of fibres containing alpha-actinin containing premyofibrils, the construct actc1b-actn2-mKate2 was injected into one-cell stage embryos as described. Whole-mount larval zebrafish were labelled using standard immuno-histochemical procedures to determine expression and localisation of different proteins.^38^ In brief, following whole mount fixation, zebrafish were incubated in blocking solution (10% fetal calf serum (Sigma) and 1% DMSO (Sigma) within PBT for 30 min at room temperature. Primary antibodies were diluted in blocking solution and samples were incubated overnight at 4 °C and washed in PBT. For imaging, embryos were mounted in 0.5% agarose on a silicon mold made using a TU-1 microinjection mold (Adaptive Science Tools) and images were taken on LSM 710 confocal (Carl Zeiss Microimaging). In determining p-FAK levels at the MTJ, the primary antibody used was p-FAK (Invitrogen, 1:50). The secondary antibody used was AlexaFluor633 (Invitrogen, 1:500). To quantify the expression levels of p-FAK at the myoseptum, imageJ (Fiji) was used to determine the mean expression level for each previously determined area surrounding the myoseptum, and these data were analysed using a Student’s unpaired two-tailed *t* test (GraphPad prism). When comparing multiple samples, ANOVA with Tukey’s post-hoc analysis was used.

### Muscle force measurements

Larvae 6 dpf were immobilised in 0.0013% tricaine, then mounted horizontally using two aluminium clips onto an in vitro apparatus (model 1500A, force transducer 403A, Aurora Scientific, Ontario, Canada). The preparations were held in a MOPS buffered physiological solution at 22 °C and stimulated via two platinum electrodes. The optimal length for active contraction was first determined, then muscles were stimulated at this length at supramaximal voltage for maximal force production. For analysis, the active force measured at each pulse duration was normalised to the active force at 5 ms.

### Delivering laminin-111 protein intramuscularly

Zebrafish 72 hpf were anaesthetised in 0.0013% tricaine. Zebrafish were mounted in a silicone cast mould with 0.5% agarose (Sigma). Laminin-111 protein (Invitrogen, Engelbreth-Holm-Swarm murine sarcoma basement membrane) was fluorescently labelled with Alexa Fluor 546 dye using a kit (Invitrogen) as per the manufacturers’ instructions and previously published techniques.^63^ In brief, 2 mg/ml laminin in PBS was added to a vial of reactive dye and stirred for 1 h at room temperature, and then passed through a column containing Bio-Rad P30 purification resin and eluted in buffer. The eluted buffer was collected as fractions and the dye containing fraction was determined with a hand-held UV lamp. Subsequently, fluorescently labelled Laminin-111 at 250 μg/ml was microinjected using a glass capillary microinjection needle and a screw-actuated micrometre driven Hamilton syringe. Borosilicate filament-injected needles (1.0 mm outer diameter, 0.78 mm inner diameter and 150 mm length, Harvard Apparatus) were prepared with a needle puller (Kopt needle/pipette puller, Model 730, David Kopf Instruments). Microinjection was performed using a glass capillary microinjection needle with IM 300 Microinjector (Narishige) and a screw-actuated micrometre driven Hamilton syringe under a Zeiss Stemi SV6 dissecting microscope (Carl Zeiss Microimaging). A volume of 1 nl was injected into larval zebrafish, and the level of fluorescence monitored. Using this approach, fluorescently labelled Laminin-111 protein was delivered focally into the myotome at levels that approximate that present in the laminin-enriched areas of the myotome in uninjected animals. Following microinjection, animals were recovered from anaesthetic and maintained in standard embryo media.

### Laminin stem cell treatments

Heterozygous *lama2;* TgBAC(pax3a:GFP)^+^ zebrafish were crossed with heterozygote *lama2* zebrafish and their progeny were screened for green fluorescence and then at 3 dpf they were also screened for birefringence to identify *lama2* homozygotes. The screened larval zebrafish were then intramuscularly microinjected with a laminin protein treatment or PBS at 3 dpf and a control group of zebrafish were also left untreated. Three days post-injection (6 dpf) zebrafish were imaged on the confocal with a minimum of four zebrafish imaged per treatment. Once all zebrafish have been imaged, they were genotyped to confirm that the visual screening of the zebrafish was correct. All pax3-GFP^+^ cells were counted per myotome within a previously determined area (within imaris, images were analysed within an area, *x* = 253 μm, *y* = 512 μm and *z* = max, this was generally an area of 1.5 myotomes. Total number of cells was divided by 1.5 to get a final number of cells per myotome) and Prism6 was used to perform statistical analysis.

### qPCR analysis

RNA was extracted from larvae or cells using a Trizol protocol. A total of 5 μg of RNA was mixed with 10 mM dNTP mix and 250 ng of random primers and heated to 65 °C for 5 min. The reaction was cooled on ice for a minute and added first strand buffer, 0.1 M DTT, RNaseH Recombinant RNase inhibitor (40 units/μl) and incubated at 50 °C for 1 h. The reaction was inactivated by a 70 °C incubation for 15 min. cDNA concentration was gauged on a nanodrop and 20 ng cDNA samples were run in triplicate with LightCycler® 480 SYBR Green I Master reaction mix (Roche) and primers as below. Reactions were run using the Roche LightCycler® 480 Real-Time PCR Machine. CT values for each sample set were normalised against the housekeeper genes, before comparing the datasets. Dmd: Forward (F) CAGTAGAGAGGGAACACCGC, Reverse (R) ATCTCGCCTCCCAGAAAACG. Dmd2: F GATTCGCCCCCAAAACAACA, R GTTCTCCCTCATCTCGCCTC. Housekeeping genes. βactin: F CGAGCTGTCTTCCCATCC, R TCACCAACGTAGCTGTCTTTCTG. zrp113a: F TCTGGAGGACTGTAAGAGGTATG, R AGACGCACAATCTTGAGAGCAG. Ef1α: F GCTGGCAAGGTCACAAAGTC, R GAACACGCCGCAACCTTTG.

### Ethics

All experimental procedures were reviewed and approved by Monash Animal Services Ethics Committee prior to commencement.

### Reporting summary

Further information on research design is available in the Nature Research Reporting Summary linked to this article.

## Supplementary information

Supplemental Data

Supplementary Movie 1

Supplementary Movie 2

Supplementary Movie 3

Supplementary Movie 4

Supplementary Movie 5

Supplementary Movie 6

Supplementary Movie 7

Supplementary Movie 8

Supplementary Movie 9

Reporting Summary Checklist

## Data Availability

The datasets generated in the current study are available from the corresponding author on reasonable request.
