# Supplementary material for: Cellular rescue in a zebrafish model of congenital muscular dystrophy type 1A
Source: NPJ Regen Med. 2019 Nov 15;4:21. doi: 10.1038/s41536-019-0084-5 (PMC6858319; doi:10.1038/s41536-019-0084-5)
Supplement: Supplementary file 1 — Supplemental Data [file 41536_2019_84_MOESM1_ESM.pdf]

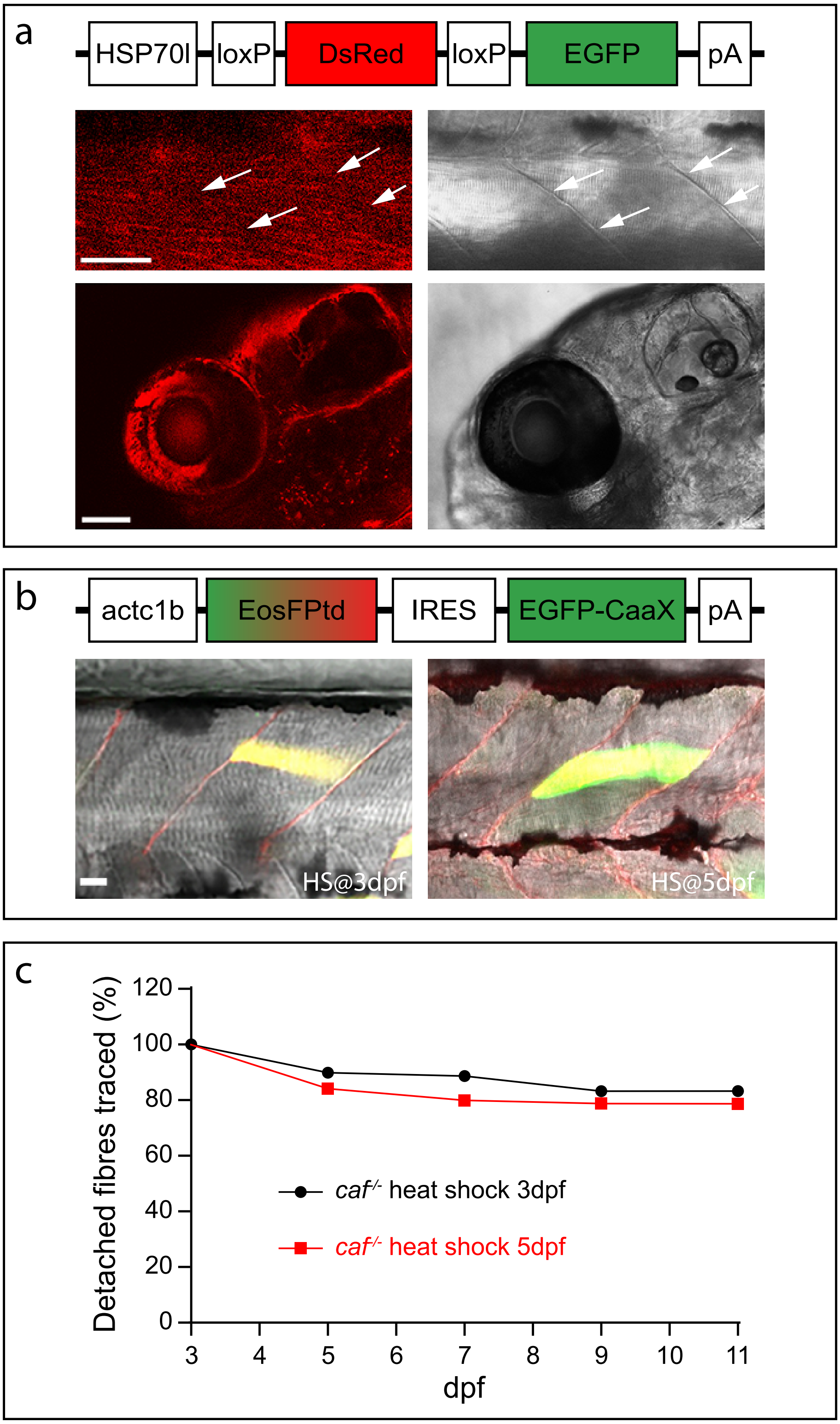

Supplementary Figure 1. Heat shock rescue controls and fibre survival analyses.
a. Expression of DsRed from a heatshock promoter does not result in localization of DsRed to the myotomal basement membrane. In this instance, a floxed DsRed construct is used which, in the absence of Cre only DsRed is expressed. Arrows mark the myosepta in the upper panels (Scale Bar, 150µm). General expression in the head of the larvae is illustrated below (Scale Bar 100µm). Left hand panels reveal DsRed expression and right hand panels are bright field images. n=10 fish examined, 5dpf larvae shown. b. Individual fibres marked by mosaic expression of actc1b:EosFPtd-IRES-EGFP-CaaX were photoconverted in *lama2*$^{-/-}$ larvae transgenic for Tg(hsp90:Lama2-mCherry) heat shocked at 3 and 5dpf. Scale Bar, 20µm. c. Individual fibers were tracked for survival until 11dpf and plotted as a percentage of original labelled fibers.

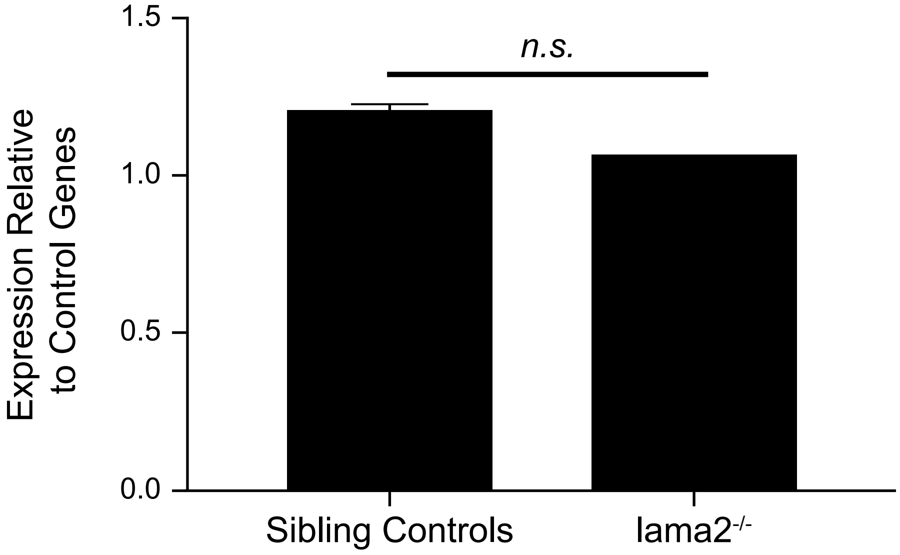

Supplementary Figure 2. Dystrophin mRNA expression levels in *lama2*$^{-/-}$ mutants.
qPCR analysis reveals no difference in levels of dystrophin mRNA expression between *lama2*$^{-/-}$ mutant larvae and siblings. One-way ANOVA, Tukey's test. Results are expressed as a mean ± SD.

Supplementary Movie 1. Remodelling of dystrophic fibres in *lama2*$^{-/-}$ larvae. Mosaically labelled act1b:GFP dystrophic fibres in *lama2*$^{-/-}$ larvae reveal extensive remodelling and reattachment to the matrix rich regions of the vertical myosepta. Maximum projection images of consecutive confocal z-stacks.

Supplementary Movie 2. Expression of the transgenes Tg(actc1b:EBFP2)^{pc5} and GT(dmd-citrine)^{ct90a} in wildtype siblings. Maximum projection images of consecutive confocal z-stacks.

Supplementary Movie 3. Expression of the transgenes Tg(actc1b:EBFP2)pc5 and GT(dmd-citrine)^{ct90a} in *lama2*$^{-/-}$ mutants. Maximum projection images of consecutive confocal z-stacks.

Supplementary Movie 4. Hyper-fusion in *lama2*$^{-/-}$ dystrophic fibres. Continuous z-stack image of hyper-fused fibres in the dual transgenic line Tg(actc1b:GFP-CaaX) and Tg(actc1b:h2afv-mCherry).

Supplementary Movie 5. Hyper-fusion does not occur in wildtype sibings. Confocal z-Stack of a wild-type 3dpf larvae dual transgenic for Tg(cmet:KalTA4-2A-mCherry; UAS-nlsGFP) and Tg(actc1b:BFP) where green marks the cmet positive satellite cell compartment and blue differentiated muscle fibres.

Supplementary Movie 6. Hyper-fusion is derived from the muscle stem cell compartment. Confocal z-Stack of a *lama2*$^{-/-}$ 3dpf larvae dual transgenic for Tg(cmet:KalTA4; UAS-nlsGFP) and Tg(actc1b:BFP) where green marks the cmet positive satellite cell compartment and blue differentiated muscle fibres. The GFP positive, satellite cell derived nuclei occur specifically in *lama2*$^{-/-}$ dystrophic fibers.

Supplementary Movie 7. Animated maximum projection image of the larva illustrated in Fig. 3C in which a *lama2*$^{-/-}$ mutant, transgenic for Tg(hsp90l:Lama2mCherry) upon heat shock expresses the Lama2-mCherry fusion protein (red) and rescues the *lama2*$^{-/-}$ pathology. Blue marks differentiated muscle fibres in the Tg(actc1b:BFP). Green marks the membrane of all cells via a ubiquitous GFP-CaaX over expression. The green channel was not included in the image in Fig. 3C for clarity. The focal “dot-like” Lama2-mCherry localisation results from the global heat shock strategy, which also induces expression in non-myotomal cells (most prominent in cells of the epidermis).

Supplementary Movie 8. Animated maximum projection image of the larva illustrated in Fig. 3B. Germline transgenic expression of Tg(actclb:Lama2-mCherry) results in correct Lama2-mCherry (Red) deposition at the myosepta in *lama2*$^{-/-}$ deficient (B) larvae . Blue marks differentiated muscle fibres using the Tg(actc1b:BFP) transgene and reveals the rescue of the dystrophic phenotype.

Supplementary Movie 9. Animated confocal stack of the larva illustrated in Fig. 3C in which a *lama2*$^{-/-}$ mutant, transgenic for Tg(hsp90:Lama2-mCherry) upon heat shock expresses the Lama2-mCherry fusion protein (red) and rescues the *lama2*$^{-/-}$ pathology. Blue marks differentiated muscle fibres in the Tg(actc1b:BFP). Green marks the membrane of all cells via a ubiquitous GFP-CaaX over expression. Note that the punctate staining of Lama2-mCherry that results from the global heatshock is most promi-nent in the epidermis.
